# Highly Sensitive Persons during War – The Case of Israeli Adults after the October 7, 2023, Terror Attack

**DOI:** 10.1192/j.eurpsy.2026.11469

**Published:** 2026-07-31

**Authors:** A. Goldberg

**Affiliations:** Education & Psychology, Tel-Hai Academic College, Kiryat-Shmona, Israel

## Abstract

**Introduction:**

Highly sensitive persons (HSPs) possess a temperament marked by an enhanced capacity to deeply process both internal states and external stimuli. This trait is associated with increased vulnerability to a range of psychological and physical challenges, including heightened stress responses

A substantial body of research indicates that HSPs are particularly responsive to their environments, both positively and negatively. This heightened environmental sensitivity means that their experiences tend to have a stronger impact on personality development and emotional functioning. In nurturing and supportive contexts, high sensitivity has been linked with psychological resilience, lower levels of aggression and fewer behavioral issues, enhanced outcomes from therapeutic and educational interventions, and improved mental health. However, recent findings also suggest that HSPs may show stronger emotional responses to subjectively negative daily experiences, with no corresponding increase in reactivity to positive events. Therefore, the nature of everyday stressors` influence on the well-being of HSPs needs further investigation.

**Objectives:**

The current research focused on highly sensitive persons (HSPs), who constitute 20% to 30% of the population and have been found to be disproportionately susceptible to environmental factors (Pluess et al., 2023). It sought to discover whether the responses of HSPs might be more acute than other individuals regarding extremely traumatic experiences such as war and displacement, specifically as to the element of collective trauma.

**Methods:**

Participants were 410 Israeli adults, who completed self-report questionnaires in two phases. First, they completed the Highly Sensitive Person Scale – Brief Version (HSP-12)**,** the Experiences in Close Relationships-Relationship Structures Questionnaire (ECR-RS), and the Mental Health Continuum Short Form (MHC-SF). Then they were asked to listen to a certain Israeli song that had become a prayer and hope for the hostages coming back home and to fill out the Positive and Negative Affect Schedule (PANAS).

**Results:**

Results showed negative association between HSP and well-being and positive association with negative affect during exposure to the song.

**Conclusions:**

These findings indicate worse outcomes for HSPs under highly stressful conditions and point to the need for further research, specifically longitudinal research, within mental health care regarding individual differences in temperament and the need for screening individuals for high sensitivity for better use of clinical interventions.

**Disclosure of Interest:**

None Declared

